# Optimal choice of functional and off-target effect-reduced siRNAs for RNAi therapeutics

**DOI:** 10.3389/fgene.2013.00107

**Published:** 2013-06-11

**Authors:** Kumiko Ui-Tei

**Affiliations:** ^1^Department of Biophysics and Biochemistry, Graduate School of Science, The University of TokyoTokyo, Japan; ^2^Department of Computational Biology, Graduate School of Frontier Sciences, The University of TokyoChiba, Japan

## Introduction

Small interfering RNA (siRNA) represses a specific target gene that has a perfectly complementary sequence by a pathway referred to as RNA interference (RNAi). Researchers have successfully diverted this natural pathway to artificially knockdown intended genes. RNAi shows promise for therapeutic applications to human diseases (Castanotto and Rossi, 2009; Ketting, 2011). Over the past decade, more than twenty siRNAs have been applied to clinical trials for dozens of diseases, including various cancers, viral infections, and genetic disorders (Davidson and McCray, 2011; Snead and Rossi, 2012). Ongoing patient trials are expected to yield success. However, several major problems, such as the efficacy and off-target effect of siRNA, remain to be overcome before RNAi-based therapeutics can be efficiently introduced into clinical practice. In the present paper, I propose a procedure for selecting sequences of highly functional siRNAs with few off-target effects.

## Functional siRNAs

**Figure 1:**
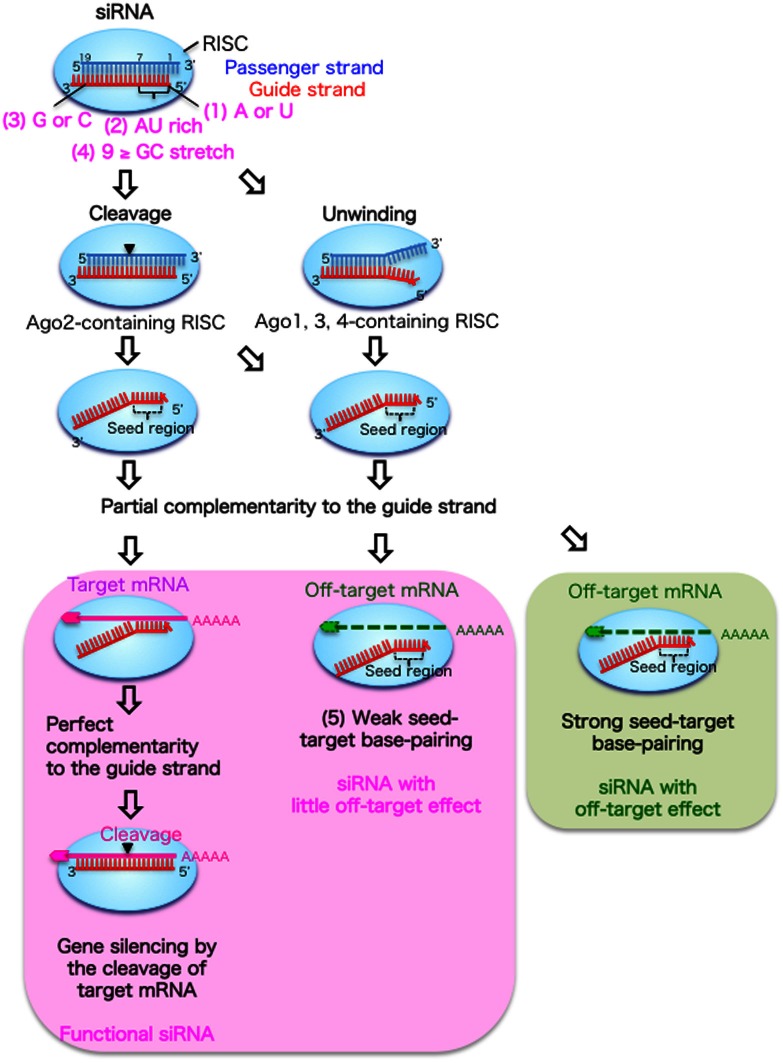
**Functional siRNA sequence and the mechanism of RNAi without off-target effects in mammalian cells**. An RNA strand with A or U at position 1 measured from the guide strand 5′ end (1), four to seven A/Us in positions 1–7 (2) and G/C at position 19 (3) are easily unwound from the 5′ end and retained in the RISC. The passenger strand is cleaved in the Ago2-containing RISC, but dissociated from the Ago1, 3, or 4-containing RISC following unwinding. In the RNAi pathway (left column), the guide strand recognizes target mRNA with completely complementary sequences, and the target mRNA is cleaved by the Ago2 protein. However, off-target transcripts with partial complementary sequences to the seed region positions 2–8 are downregulated or not downregulated according to the thermodynamic stability of the duplex formed between the siRNA seed region and target mRNA. The siRNAs with weak seed-target base-pairing are not reduced by the off-target effect (middle column), but those with strong base-pairing are downregulated by the off-target effect (right column).

In RNAi experiments, siRNAs composed of duplexes of 21-nucleotide (nt) RNA with 2-nt 3′-overhangs are usually used. As shown in Figure 1, siRNAs that are incorporated into cells are transferred to an RNAi effector complex called the RNA-induced silencing complex (RISC) (Hutvagner and Simard, 2008; Jinek and Doudna, 2009), which contains the Argonaute 2 (Ago2) protein with slicer activity (Hammond et al., 2001; Martinez et al., 2002). In the RISC, the siRNA duplex is unwound into single-stranded RNAs. The guide strand selectively retained on the RISC base-pairs with the target mRNA. Then, the target mRNA is silenced by cleavage by Ago2 protein at the region corresponding to the nucleotide positions 10–11 of the siRNA guide strand (Elbashir et al., 2001; Hammond et al., 2001; Martinez et al., 2002).

However, the efficacy of RNAi in mammalian cells varies considerably depending on the siRNA sequence, and a limited proportion of siRNAs is functional (Holen et al., 2002; Harborth et al., 2003). We and others have reported that preferable incorporation of the siRNA guide strand into the RISC is due to the asymmetrical features of both siRNA terminals (Amarzguioui and Prydz, 2004; Reynolds et al., 2004; Ui-Tei et al., 2004). The siRNA guide strand, which has a thermodynamically less stable 5′-end, is preferentially retained on the RISC (Khvorova et al., 2003; Schwarz et al., 2003; Ui-Tei et al., 2004). In contrast, the passenger strand is cleaved by the Ago2 protein on the RISC and degraded (Matranga et al., 2005; Miyoshi et al., 2005; Rand et al., 2005; Leuschner et al., 2006). We extensively examined the relationship between siRNA sequence and its ability to give rise to RNAi in mammalian cells (Ui-Tei et al., 2004), and empirically defined the practical characteristics of highly functional siRNAs as follows: (1) A or U residues at nucleotide position 1; (2) four to seven A/Us at nucleotide positions 1–7; and (3) G/C at position 19, with the nucleotide position measured from the 5′-end of the guide strand (Figure 1). In addition, (4) a GC stretch of no more than 9 nucleotides should occur in the siRNA sequence. Among these rules, Reynolds et al. (2004) also showed that A is preferable at position 1 and not G/C, and Amarzguioui and Prydz (2004) also recommended A/U but not G at this position. Furthermore, Amarzguioui and Prydz also recommend G/C but not U at the position 19.

All three features from (1) to (3) may be involved in the thermodynamic asymmetry of both terminals in the selection of the functional guide strand RNA. However, feature (1) should also be involved in the other property that strongly contributes to the RNAi activity. The 5′-end of the siRNA guide strand is reported to be anchored in the binding pocket of the Mid domain of the *Archaeoglobus fulgidus* Ago-like protein (Ma et al., 2005; Parker et al., 2005). The crystal structure of a MID domain of human Ago2 and NMR titration experiments showed that nucleotide monophosphates, AMP and UMP, bind with up to 30-fold higher affinities than either CMP or GMP, providing structural evidence for preferential interactions of the A/U residues in the MID domain of eukaryotic Ago proteins (Frank et al., 2010). In the RNAi pathway, thermodynamic asymmetry due to features (2) and (3) may be essential for dissociating double-stranded siRNA into single-stranded guide RNA from its 5′-terminal. Then, the 5′-end of the siRNA guide strand should be anchored in the Ago2 pocket due to feature (1), immediately after unwinding, since the passenger strand in the siRNA duplex is cleaved by the catalytic activity of Ago2 protein and degraded (Figure 1) (Kawamata et al., 2009; Yoda et al., 2010). However, in the case of siRNA with long GC stretches, the 10-nt and 11-nt fragments of the passenger strand cleaved by Ago2 cannot be dissociated from the guide strand due to the strong base-pairing by the long GC stretch. This may explain why feature (4) is a preferable condition for a functional siRNA.

## Mechanism to avoid seed-dependent off-target effects

RNAi has been assumed to be an extremely specific procedure to knockdown a particular target gene. However, accumulated evidence from genome-wide experiments has revealed that numerous non-targeted mRNAs with partially complementary sequences in their 3′-UTRs to the guide strand seed region (positions 2–8) are also reduced (Jackson et al., 2003, 2006; Lin et al., 2005; Birmingham et al., 2006; Ui-Tei et al., 2008) through a mechanism similar to that of miRNA silencing (Lewis et al., 2005; Lim et al., 2005; Grimson et al., 2007). This phenomenon is referred to as the off-target effect, and it is likely to be due to the fact that the seed region is situated on the surface of Ago in a quasi-helical form, serving as the entry or nucleation site for small RNAs in the RISCs (Ma et al., 2005; Yuan et al., 2005). In the pathway of the off-target effect, most of the siRNA duplexes may be loaded onto Ago proteins other than Ago2, such as Ago1, 3, and 4, which lack slicer activities (Figure 1). Thus, the thermodynamic asymmetry of both terminals due to features (1)–(3) should be essential for siRNA unwinding, as the passenger strand in the siRNA duplex is not cleaved by Ago2 protein, in contrast to the case of RNAi.

The off-target effect is induced by only 7-nt base-pairing between the siRNA seed region and the mRNA 3′-UTRs. If such 7-nt base-pairing is a unique factor to induce the off-target effect, a target gene-specific RNAi is not possible, since random 7-mer sequences are stochastically distributed at about ≥1000 sites in 3′-UTR regions. Comparing the efficiencies of off-target effects using various reporter plasmids with different 7-nt seed sequences, we found that the ability to induce the off-target effect strongly correlated with the thermodynamic stability of the duplex formed between the siRNA seed region and target mRNAs (Ui-Tei et al., 2008, 2012). This result was confirmed by genome-wide expression profiling using siRNA with high or low seed-target stability (Ui-Tei et al., 2008). It was revealed that siRNA with high stability (benchmark melting temperature [*T*_m_] ≥ 21.5°C) in the 7-bp duplex is a good inducer of off-target activity, whereas siRNA with low stability in the 7-bp duplex (*T*_m_ ≤ 21.5°C) is a poor inducer. Approximately 22% of the 16,384 sequences of 7-nt had *T*_m_-values ≤ 21°C, indicating that limited seed sequences are available for selecting siRNAs with reduced off-target effects.

It was shown that siRNA off-target effect is induced by a similar mechanism to miRNA-mediated gene silencing (Lewis et al., 2005; Lim et al., 2005; Grimson et al., 2007). Thus, it was expected that miRNA silencing efficacy is also determined by the thermodynamic stability in the duplex formed between the miRNA seed region and the target mRNA. We carried out reporter experiments using various miRNAs to evaluate their silencing efficacies. However, unlike the siRNA off-target effect, the silencing efficacy of miRNA was not simply determined by seed-target stability, but also affected by the instability of the 5′ region of the miRNA duplex (Hibio et al., 2012). MiRNAs with an unstable 5′ terminal duplex and stable seed-target duplex exhibit strong silencing activity. The RNA strand in a small RNA duplex containing the thermodynamically less stable 5′-end is preferentially loaded onto the RISC (Khvorova et al., 2003; Schwarz et al., 2003; Ui-Tei et al., 2004). Although siRNA is simply composed simply of perfectly complementary double-stranded RNA, miRNA has specific structural features, such as an internal bulge/mismatch. Because the 5′ region and seed region overlap each other, the stabilities of these regions in siRNA are similar. These regions in siRNA may function in a similar manner, and the stability of seed region alone is enough to define the efficacy of siRNA off-target effect. However, the efficacy of miRNA-mediated silencing activity may be defined by the combination of the nucleotide sequence in the seed region and structure of 5′ region of miRNA duplex. Although the structure depends directly on the nucleotide sequence, the sequence and the structure may contribute to silencing efficacy as distinguishable parameters.

## Conclusion and optimal choice of functional siRNA with reduced off-target effect

For safe RNAi therapeutics, it is important to select highly functional siRNAs that have reduced off-target effects. I propose that those siRNAs that simultaneously satisfy the following five conditions should be good candidates:

1. A or U residues at nucleotide position 1 measured from the 5′-end of the guide strand
2. Four to seven A/Us in nucleotide positions 1–7 (A/U ≥ 57%)
3. G/C at position 19
4. No long GC stretch (≤9 GC)
5. Low thermodynamic stability in the duplex formed between the siRNA 7-nt seed region and the target mRNA (*T*_m_ ≤ 21.5°C)

To facilitate the selection of such siRNAs, we constructed the siRNA design webserver siDirect 2.0 (http://siDirect2.RNAi.jp/) (Naito et al., 2009; Naito and Ui-Tei, 2012). siRNAs for >94% of human mRNA sequences in RefSeq can be successfully designed using our procedure.
